# Commentary

**DOI:** 10.1186/1617-9625-1-13

**Published:** 2002-06-15

**Authors:** ED Nelson

## 

Ed Nelson, Sc.D., founder of the International Society for the Prevention of Tobacco Induced Diseases (PTID) and founding editor of this journal, died unexpectedly July 11 in Essen, Germany. His death leaves a major gap in international efforts to combat the various diseases that plague mankind due to tobacco addiction and exposure.

An internationally regarded toxicologist, Ed was born in the United States. At the time of his death he was a professor at the Essen University School of Medicine, where he earned his Med.B. in 1985, and his Sc.D. in 1989.

Throughout his career, Ed dreamed of a strategy to combat smoking, including passive smoking. After undergoing a heart transplantation in 1993, he intensified his plans to build an international society for the prevention of tobacco induced diseases. With all his enthusiasm, he worked to make this dream come true, founding PTID in December 2000. He worked days and nights, not only as the founder and president of the society, but functioning as his own secretary and manager, and publishing the society's journal, *Tobacco Induced Diseases*. He wanted PTID to become an organization of the highest class, and he was determined that tobacco industry interests were not to influence PTID in any way. He was very serious with membership applications because he wanted only the best researchers involved in his plans.

In April 2002, Ed knew that he would need a second heart transplantation, but he did not regard that as reason for resignation. Quite the reverse: He worked to organize the PTID-Society's first annual scientific meeting, to be held in Essen, for October 2002. He was not sure it would happen, but he planned as if it would happen, hoping that if he received a new heart and successfully underwent the transplantation, he could be present to start the meeting. Nevertheless, Ed was very realistic. While planning, he prepared letters for the society members, to be sent in the event of his death, and made a request to all members: PTID should survive; it should grow and prosper. That was Ed's greatest wish until he died.

The conference was indeed held. Perhaps the greatest testimony to Ed's memory and his influence is that people flew to the meeting from all parts of North America, Asia, and Europe. His dream was realized and will continue to be realized in the work of PTID. We are sure that Ed is carefully watching over all of us.

His wife, Anke, and children, Shirley and Scott, survive Ed. We thank them for letting him establish PTID and hope that his memory will provide them comfort and joy.

Daniel R. Longo, Sc.D.

Editor

## Competing interests

The authors declare that they have no competing interests.

